# Asthma Patients' Use of Cell Phone Features and Their Willingness to Use Them for Self-Management

**DOI:** 10.7759/cureus.23292

**Published:** 2022-03-18

**Authors:** Rawan A Alshehri, Abdullah T Alanazi

**Affiliations:** 1 Health Informatics, King Saud Bin Abdulaziz University for Health Sciences, Riyadh, SAU; 2 Health Sciences, King Abdullah International Medical Research Center, Riyadh, SAU

**Keywords:** self-management, self-care, mobile phone, asthma, mhealth

## Abstract

Introduction

Mobile health has the potential to improve self-care for people with chronic conditions. There are few previously published studies that have examined asthma patients' use of cell phone features and their willingness to use them for self-care in Saudi Arabia. Moreover, there is no validated instrument in Arabic to test the use of cell phone functions.

Aim

The aim of this study was to investigate the general use of cell phone features by asthma patients and their willingness to use them and to determine the frequency of use of cell phone features by asthma patients. It also aimed to translate and validate an Arabic version of the questionnaire to test the use of cell phone functions by asthma patients.

Methods

This study was conducted in Saudi Arabia in 2021 using an online questionnaire. The test was translated (changes were made as needed) and validated using Cronbach's alpha coefficient. After validation of the instrument, the questionnaire included 34 questions divided into three sections: demographic information, current use of cell phone features, and desire to use cell phone features. Adults with asthma aged 18 years and older who owned a smartphone participated in the study. Data were analyzed using descriptive statistics.

Results

Two hundred and seventy asthma patients completed the questionnaire. The Cronbach's alpha value of the questionnaire was 0.86, indicating its reliability and consistency. Only 39 (14.4%) of the participants use cell phones for asthma care, and 13 (4.8%) have apps for asthma care on their cell phones. However, 74.1% of participants have a desire to use a cell phone for asthma care. The majority of participating asthma patients (67.8%) occasionally used the mobile internet browser to search for asthma information. Surprisingly, most of the participating asthma patients did not show a greater desire to use methods other than the internet browser to obtain asthma information. They did, however, have a strong desire to use the news to obtain information about asthma.

Conclusion

Because the study shows that asthma patients prefer internet searches followed by the news to obtain information, stakeholders need to pay more attention to the quality, quantity, completeness, and accuracy of health information in these media.

## Introduction

Asthma is the most common chronic inflammatory lung disease, affecting more than 339 million people worldwide in 2016 [1,2]. Statistics provided by the World Health Organization (WHO) indicated that there were 417,918 deaths from asthma worldwide in 2016 [3]. Asthma has a negative effect on patients, their relatives, and society by decreasing quality of life, requiring constant medical services (emergency departments, doctor visits, etc.), and increasing hospitalizations. Saudi Arabia (SA) is a very large Asian country and the second-largest country in the Arab nation. It has a total population of about 31.54 million people with an average age of 71 years. It is noted that in SA, the number of people suffering from asthma is greater than two million. According to the Ministry of Health, the prevalence of asthma in SA is between 15% and 25% [1]. Despite an evidence-based approach to the disease and various treatment options, asthma is still uncontrolled. Patients with asthma who take good care of themselves typically benefit from fewer hospitalizations, improved lung function, fewer symptoms, and better adherence to treatment. One solution for promoting self-care skills in patients with asthma is to provide self-care opportunities, such as providing basic knowledge about the nature of asthma, prevention of allergen sensitivity and its causes, treatment with medications, delivering alerts and reminders to patients, and demonstrating the use of therapeutic agents through the use of information technology tools (e.g., internet and cell phones) [2]. The use of cell phones and other wireless technologies to improve the delivery of health-related facilities is part of mHealth. According to WHO, mHealth is defined as "medical and public health practices supported by mobile devices such as cell phones, patient monitoring devices, personal digital assistants, and other wireless devices" [4]. However, there is a lack of data on the impact of mHealth on asthma self-care.

In Taiwan, researchers analyzed the impact of using a self-treatment method for chronic asthma on 120 patients who visited outpatient clinics. Participants were randomly assigned to one of two groups: a mobile phone-based integrated plan or a control group. This study showed that a dedicated cell phone-based self-care system could help improve asthma control and quality of life while reducing the number of exacerbations and unscheduled appointments (p<0.05) [5]. Another systematic review conducted in 2017 investigated whether mobile health apps can help asthma patients properly control their disease. Most of the studies conducted (9/10, 90%) showed that mHealth applications for multifunctional use could help asthma control and were also associated with an improvement in lung capacity and quality of life [6]. Recently, researchers conducted a study of 408 people with asthma to investigate the efficacy of an online method to help control asthma. In a one-year blinded randomized clinical trial, digital alerts sent to patients resulted in better asthma control [7].

Nevertheless, in 2008, a group of 288 people in the United Kingdom who had difficulty controlling their asthma were randomly assigned to one of two groups to determine whether smartphone-based screening techniques improved asthma management as compared with traditional paper-based approaches. The result of that study indicated that the increase in both asthma regulation and self-efficacy was not statistically significant [8]. Understanding patients' behavior in using such technologies is one of the most important elements for the effective adoption of mHealth.

In a survey of 3,336 individuals conducted in the United States, researchers examined patients' views of using technology to connect with their primary care teams. They also examined their behaviors related to using new technologies to communicate with other patients. More than half of the participants reported using Facebook (58%) and text messaging (64.1%). Compared with social media (3.1%) and text messaging (13.3%), telephone contact and email were methods preferred by participants for communicating health-related goals (75.5% and 48.8%, respectively). Moreover, peer counseling via Facebook (11.7% of participants) was significantly more common among younger participants (p<0.0001), women (p<0.001), and minority groups (p<0.0004) [9].

In another study conducted in the United Kingdom, the authors administered a questionnaire to randomly selected general practitioners, asthma nurses, and individuals with asthma (12 years and older) to explore attitudes regarding the use of electronic self-monitoring. The results indicated that patients and medical providers appreciated the technology. The primary complaints of both physicians and patients were the cost and time involved [10].

In addition, a study conducted in the United States surveyed the use of electronic media and users' concerns about using electronic media to interact with healthcare professionals regarding asthma. The most common choice for accessing information about asthma was email. The data indicated that many respondents felt that social media was primarily for communicating with peers. In addition, privacy issues were also raised [11].

In studies completed in Iran in 2018, a research group enrolled 146 patients with asthma to investigate their use of cell phone features and their willingness to use these features for self-care. Their results indicated that many people with asthma use internet searches for educational purposes and are willing to use social media for self-care. Cell phones were considered suitable for accessing instructional knowledge and notifications [2].

In SA, a cross-sectional survey was conducted in 2012 among 83 patients with asthma in the respiratory outpatient clinic of the Security Forces Hospital in Riyadh. That study aimed to determine the online knowledge needs of patients with asthma and analyze the associated causes. The results revealed that two-thirds of the respondents using the internet had searched for asthma knowledge, including side effects of treatment and complications. Of the respondents, 78% wanted an asthma knowledge base in Arabic. White-collar (p=0.003), more highly educated (p=0.004), and higher-earning patients (p=0.005) use the internet as a preferred source of additional knowledge about asthma [12]. In Saudi Arabia, there is no evidence regarding the usability of phone features for self-care among asthma patients. This study aims to investigate the general use of and willingness of patients with asthma to use cell phone features and to determine the frequency with which these patients use cell phone features.

## Materials and methods

In 2021, a multi-center descriptive cross-sectional study was conducted in Saudi Arabia. That study used a self-administered questionnaire to investigate how patients with asthma use and want to use the features of cell phones to promote self-care. Study participants were patients aged 18 years or older who were clinically diagnosed with asthma. In this regard, researchers excluded subjects who did not read Arabic. A total of 377 patients was required to obtain a 95% confidence interval. The data collection method of the study included the use of a questionnaire that was validated by a previous study [2]. The translation and validation of the questionnaire were done according to the following method:

1. Two forward translations for the English version of the questionnaire were performed by two independent translators who were bilingual native Arabic speakers to obtain the forward versions (T1 and T2).

2. The two forward versions (T1 and T2) were merged in the first step by the two translators, with a third bilingual person or more mediating a discussion between them to synthesize the results of the translations and obtain the forward version T-12.

3. Two back-translations of the Arabic consensus translation were performed by two independent translators who did not know the original English questionnaire, were not involved in the forward translation, and were preferably native English speakers to obtain the back-translations BT1 and BT2.

Subsequently, all translations were evaluated by experts to reach a consensus on any discrepancies to produce a preliminary version of the questionnaire and to check the face and content validity (changes were made as needed). The Arabic version was then tested by ten native Arabic speakers to ensure that it was readable and understandable. The questionnaire was randomly sampled and distributed to 468 potential participants; only 270 asthma patients were willing to participate and complete the questionnaire.

Before the data obtained from the questionnaire were analyzed, we evaluated the reliability using Cronbach's alpha coefficient, which had to exceed 0.7 to be considered reliable. After instrument validation, the final questionnaire included 34 questions divided into three major sections: demographic information (five questions), overall use of cell phone functions (18 questions), and desire to use cell phone functions (11 questions). The study cover sheet informed participants of the purpose of the study, and informed consent was obtained.

## Results

Cronbach's alpha value for the translated questionnaire was 0.86, indicating that the questionnaire was reliable and consistent. The questionnaire was distributed to 468 potential participants (asthma patients) in three regions in Saudi Arabia. However, only 270 individuals were willing to participate in the study and completed the questionnaire, representing a response rate of 58%. Out of the 270 asthma patients who participated, 196 (72.6%) were female. Most participants were between 18 and 29 years old (67.8%) and had a bachelor's degree (69.6%). Out of the 270 participants, 155 had asthma for longer than 11 years. In addition, Table 1 shows that 128 (47.4%) of the participants had intermittent asthma severity. Table 2 shows the frequency and percentage of participating asthma patients' use of a cell phone and their desire to use cell phones for asthma-related matters and functions. Only 39 (14.4%) participants reported using cell phones for asthma care, and 13 (4.8%) had apps for asthma care on their cell phones. However, 74.1% of the participants had a desire to use a cell phone for asthma care. Table 3 shows the frequency and percentage with which participating patients with asthma used cell phone features for obtaining asthma-related information. Most participating patients had never received asthma-related information via cell phone calls, messages, social media, emails, or apps. However, most of the participating patients with asthma (67.8%) occasionally used mobile internet browsers to search for asthma-related information. In addition, patients with asthma used social media and news at least once per week to obtain information about asthma (43.3% and 36.7%, respectively). 

**Table 1:** Demographic characteristics of patients with asthma

Demographic variable	Frequency	Percentage
Gender		
Female	196	72.6
Male	74	27.4
Age, years		
18–29	183	67.8
30–39	44	16.3
40–49	18	6.7
50–59	17	6.3
>60	8	3.0
Education level		
High school diploma or less	69	25.6
Bachelor degree	188	69.6
Higher than bachelor degree	13	4.8
Duration of asthma, years		
<5	54	20.0
5–10	61	22.6
>11	155	57.4
Severity of asthma		
Intermittent	128	47.4
Mild persistent	32	11.9
Moderate persistent	90	33.3
Severe persistent	20	7.4

**Table 2:** Overall use of and desire to use mobile phone functionality among patients with asthma (n=270)

Item	Frequency (%)	
	Yes	No
Use of mobile phones to receive asthma care services	39 (14.4)	231 (85.6)
Have mobile apps related to asthma care	13 (4.8)	257 (95.2)
Desire to use mobile phones to receive asthma care services	200 (74.1)	70 (25.9)

**Table 3:** Frequency of using mobile phone functionality among patients with asthma (n=270)

Item	Frequency (%)			
	Everyday	Several times per week	Occasionally	Never
Receiving asthma-related information through mobile phone calls (to friends, relatives, doctors, and nurses)	4 (1.5)	12 (4.4)	82 (30.4)	172 (63.7)
Receiving asthma-related information through messages (to friends, relatives, doctors, and nurses)	3 (1.1)	11 (4.1)	99 (36.7)	157 (58.1)
Using mobile internet browser to search for asthma-related information	5 (1.9)	36 (13.3)	183 (67.8)	46 (17.0)
Using social media (such as Twitter) to access asthma-related information	6 (2.2)	28 (10.4)	117 (43.3)	119 (44.1)
Using mobile email to communicate with others (friends, relatives, doctors, and nurses) to receive asthma-related information	3 (1.1)	11 (4.1)	31 (11.5)	225 (83.3)
Using of apps (software) to access asthma-related information	5 (1.9)	17 (6.3)	93 (34.4)	155 (57.4)

The results presented in Table 4 show that most of the participating asthma patients used an internet browser search to obtain information about asthma warning symptoms (21.5%), medical therapy (27.0%), allergenic and irritant substances (39.6%), peak expiratory flow (PEF) test use (29.3%), and warnings about lack of asthma control (23.3%). It also appears that most participants used social media to communicate with other asthma patients (16.7%), whereas they use messaging to receive reminders for doctor's appointments (32.6%), flu vaccination (26.7%), medication use (18.9%), and PEF testing (16.7%).

**Table 4:** Frequency of use and desire to use mobile phone functionalities for obtaining information related to asthma (n=270) PEF - peak expiratory flow

Items	Frequency (%)								
	Use								
	None		Phone/voice call	Message	Internet browser	Social media	Email	Software/apps	Video call
Receiving information about asthma warning symptoms (cough, wheezing)	120 (44.4)		19 (7.0)	24 (8.9)	58 (21.5)	43 (15.9)	2 (0.7)	2 (0.7)	2 (0.7)
Receiving information about medications	116 (43.0)		26 (9.6)	28 (10.4)	73 (27.0)	19 (7.0)	2 (0.7)	6 (2.2)	0 (0.0)
Receiving information about allergenic and irritating substances (air pollution)	86 (31.9)		25 (9.3)	31 (11.5)	107 (39.6)	14 (5.2)	1 (0.4)	5 (1.9)	1 (0.4)
Receiving information about how to use therapy aids (PEF test)	128 (47.4)		19 (7.0)	19 (7.0)	79 (29.3)	15 (5.6)	1 (0.4)	7 (2.6)	2 (0.7)
Communicating with other patients	168 (62.2)		28 (10.4)	24 (8.9)		45 (16.7)	1 (0.4)	4 (1.5)	0 (0.0)
Reminders about doctor or nurse appointments	119 (44.1)		42 (15.6)	88 (32.6)		6 (2.2)	2 (0.7)	13 (4.8)	0 (0.0)
Influenza vaccination reminder	139 (51.5)		24 (8.9)	72 (26.7)		23 (8.5)	0 (0.0)	12 (4.4)	0 (0.0)
Medication use reminder	177 (65.6)		13 (4.8)	51 (18.9)		4 (1.5)	2 (0.7)	6 (2.2)	0 (0.0)
Reminders for PEF test	190 (70.4)		21 (7.8)	45 (16.7)		6 (2.2)	2 (0.7)	6 (2.2)	0 (0.0)
Warning about lack of asthma control	142 (52.6)		22 (8.1)	30 (11.1)	63 (23.3)	10 (3.7)	0 (0.0)	2 (0.7)	1 (0.4)
Desire									
Receiving information about asthma warning symptoms (cough, wheezing)	111 (41.1)	31 (11.5)		50 (18.5)	50 (18.5)	17 (6.3)	6 (2.2)	5 (1.9)	0 (0.0)
Receiving information about medications	94 (34.8)	28 (10.4)		57 (21.1)	62 (23.0)	20 (7.4)	3 (1.1)	6 (2.2)	0 (0.0)
Receiving information about allergenic and irritating substances (air pollution)	80 (29.6)	30 (11.1)		59 (21.9)	62 (23.0)	24 (8.9)	4 (1.5)	10 (3.7)	1 (0.4)
Receiving information about how to use therapy aids (PEF test)	101 (37.4)	26 (9.6)		45 (16.7)	65 (24.1)	21 (7.8)	5 (1.9)	6 (2.2)	1 (0.4)
Communicating with other patients	147 (54.4)	30 (11.1)		37 (13.7)		44 (16.3)	5 (1.9)	4 (1.5)	3 (1.1)
Reminders about doctor or nurse appointments	105 (38.9)	32 (11.9)		92 (34.1)		18 (6.7)	7 (2.6)	16 (5.9)	0 (0.0)
Influenza vaccination reminder	130 (48.1)	23 (8.5)		73 (27.0)		29 (10.7)	6 (2.2)	9 (3.3)	0 (0.0)
Medication use reminder	146 (54.1)	27 (10.0)		58 (21.5)		16 (5.9)	6 (2.2)	17 (6.3)	0 (0.0)
Reminders for PEF test	146 (54.1)	29 (10.7)		58 (21.5)		21 (7.8)	5 (1.9)	11 (4.1)	0 (0.0)
Warning about lack of asthma control	106 (39.3)	27 (10.0)		54 (20.0)	50 (18.5)	21 (7.8)	4 (1.5)	7 (2.6)	1 (0.4)

Surprisingly, most of the participating patients with asthma showed no greater desire to use methods other than internet searches to obtain information about asthma symptoms, medical therapy, allergenic and irritant substances, and the use of the PEF test. In addition, participants did not want to use methods other than social media to communicate with asthma patients (16.3%) and receive messages to be reminded of medical appointments (34.1%), flu vaccination (27%), medication use, and PEF test (21.5%). The results in Table 5 show a significant relationship between the level of education and duration of asthma disease with patients' use of cell phone features for asthma-related services (p<0.05). However, there was no statistically significant association between age, gender, or asthma severity and patients' use of cell phone functions for asthma-related services (p>0.05). The mean and standard deviation (SD) of the total score for the use of cell phone functions for asthma-related services was 7.4±7.2. We also noted no significant relationship between the desire to use cell phone functions for asthma-related services and age, gender, education level, duration of asthma, or asthma severity (p>0.05). The mean and SD of the total score for the desired use of cell phone features for asthma-related services was 9.3±9.6.

**Table 5:** Frequency of use and willingness to use mobile phone functionalities among patients with asthma in relation to demographic characteristics * p-value less than 0.05 indicating a significant difference

Demographic variable		Use, %				Desire, %			
Gender		Low	Moderate	High	p-value	Low	Moderate	High	p-value
	Female	38 (19.5)	110 (56.4)	47 (24.1)	0.24	43 (22.1)	100 (51.3)	52 (26.7)	0.09
	Male	16 (21.3)	48 (64)	11 (14.7)		24 (32.4)	38 (51.4)	12 (16.2)	
Age, years	18–29	33 (18)	102 (55.7)	48 (26.2)	0.12	43 (23.5)	89 (48.6)	51 (27.9)	0.16
	30–39	7 (16)	32 (72.8)	5 (11.4)		10 (22.7)	26 (59.1)	8 (18.2)	
	40–49	7 (38.9)	9 (50)	2 (11.1)		7 (38.9)	9 (50)	2 (11.1)	
	50–59	5 (29.4)	10 (58.8)	2 (11.8)		5 (29.4)	12 (70.6)	0 (0)	
	>60	2 (25)	5 (62.5)	1 (12.5)		2 (25)	3 (37.5)	3 (37.5)	
Education level	High school diploma or less	6 (8.7)	49 (71)	14 (20.3)	0.03*	16 (23.5)	40 (58.8)	12 (17.6)	0.12
	Bachelor degree	47 (25)	101 (53.7)	40 (21.3)		50 (26.6)	88 (46.8)	50 (26.6)	
	Higher than bachelor degree	1 (7.7)	8 (61.5)	4 (30.8)		1 (7.7)	10 (76.9)	2 (15.4)	
Duration of asthma, years	>5	6 (11.1)	32 (59.3)	16 (29.6)	0.02*	8 (14.8)	30 (55.6)	16 (29.6)	0.08
	5–10	6 (9.8)	41 (67.2)	14 (23)		11 (18)	36 (59)	14 (23)	
	>11	42 (27.1)	85 (54.8)	28 (18.1)		48 (31)	73 (47.1)	34 (22)	
Severity of asthma	Intermittent	23 (18)	79 (61.7)	26 (20.3)	0.88	33 (25.8)	69 (54)	26 (20.3)	0.46
	Mild persistent	8 (25)	16 (50)	8 (25)		7 (21.9)	13 (41)	12 (37.5)	
	Moderate persistent	19 (21.1)	50 (55.6)	21 (23.3)		20 (22.2)	48 (53.3)	22 (24.4)	
	Severe persistent	4 (20)	13 (65)	3 (15)		7 (35)	9 (45)	4 (20)	

## Discussion

Each patient who participated in this study had a smart cell phone with internet access on their phone. Results showed that about one-third of the participants occasionally used cell phone calls to obtain asthma-related information, whereas two-thirds of the participants occasionally used their phone's internet to obtain asthma-related information. On the other hand, most participants had never used apps or emails on their cell phones to obtain asthma-related information. In addition, we found that the most frequently used cell phone function by all patients was internet search, followed by news and social media to obtain asthma-related information and to contact other asthma patients. Participating patients showed no interest in deviating from their currently used cell phone functions to obtain asthma-related information and services. Finally, this study found a significant association between education level and duration of asthma disease and patients' use of cell phone features for asthma-related services. Patients with a higher level of education were found to have a 10% higher average percentage (31%) of using cell phone features for asthma-related services (p=0.03). In addition, patients with a shorter duration of asthma (<5 years) on average used a 10% higher percentage (30%) of cell phone features for asthma-related services than did patients with a longer duration of asthma (p=0.02). A study conducted in the United States found that 64% of participating asthma patients used messaging, which is consistent with this study, in which 42% of participants reported using messaging to obtain information about asthma [9].

Two studies, one conducted in the United Kingdom and one in SA, found that asthma patients desire to use technology for asthma-related services [10,12]. These findings are consistent with those of the present study, which showed that approximately 74% of participating patients with asthma would like to use cell phones for asthma-related services. A study conducted in SA also found that patients with a higher level of education than high school frequently used the internet to learn about asthma (p=0.004). This is consistent with the results of this study, which found that a higher level of patient education was significantly related to patient use of cell phone features (p=0.03) [12]. Another study conducted in the United States found that social media was the most common method of communicating with other asthma patients, which was also found in this study. A US study also found that email was the most common choice for accessing asthma information (77%) [11]. In contrast, the use of email to communicate with others to obtain asthma-related information was low (16.7%) in this study; this might be due to concerns about patient confidentiality. In addition, providers expressed concern about the amount of time they had to spend responding to patient emails [13]. 

A 2018 Iranian study showed that the most common asthma-related function of the cell phone is internet search, which supports one of the findings of this study and proves that the most used function of the cell phone among asthma patients is internet search [2]. Moreover, the Iranian study reported that 87% of asthma patients were willing to use cell phones to access asthma services, which is consistent with the results of this study, which found that 74.1% of participants wanted to use a cell phone to access asthma services [2]. On the other hand, asthma patients showed a greater desire to use social media as compared with other cell phone features to obtain information about asthma, which is contrary to the results of our study, which indicated that most participating patients with asthma had a strong desire to use internet search. This could be because a significant proportion of the SA population (58.4%) uses internet search to find health-related information, and most users (84.2%) believe that this is a useful method of obtaining information [14].

The results of the research conducted in Iran, which found a low installation of mobile apps (16.4%), are consistent with those of the present study (4.8%). Research conducted in SA found that people with chronic diseases have low use of health apps because they do not know that health-related apps exist, they cannot use the apps due to lack of knowledge, and they consider the apps unnecessary. Consequently, efforts should be made to encourage people with chronic diseases to use health apps [15].

Strengths and limitations

This is among the first studies conducted in SA to examine the extent to which participants rely on their phone's features (i.e., phone calls, apps, messaging, internet searches, social media, and email) to obtain healthcare assistance. Moreover, this is the first study to examine the desire of patients with asthma to use their cell phones as a means to access these services for self-care.

One limitation of this study is that descriptive studies cannot be used to demonstrate causality. Another factor that can be considered a limitation is that the questions were self-administered, and respondents might have provided more socially acceptable answers. We addressed this limitation by emphasizing that the study was conducted for educational purposes only and that no personal data were collected.

Directions for future research

This study reveals the information needs and preferred functions for obtaining and using asthma-related information and services among patients with asthma. Considering that patients with asthma prefer to use their cell phone's functions to search the internet, this study could help to establish a solid asthma education website in Arabic. In addition, this study could serve as a reference for the development of future mobile health applications for patients with asthma based on their information needs. Another recommendation for future research would be to investigate the impact of using cell phone features to control asthma.

## Conclusions

In summary, asthma patients were more likely to use internet searches on their cell phones, as compared with other cell phone features, to obtain information about asthma-related concerns, such as asthma warning symptoms, medical therapy, allergenic and irritant substances, use of the PEF test, and warnings about poor asthma control. In addition, most asthma patients preferred social media to communicate with other asthma patients and used messaging to receive reminders about medical appointments, flu shots, and PEF testing. Since this study shows that patients with asthma prefer internet searches followed by the news to obtain information, stakeholders must be aware of the quality, quantity, completeness, and accuracy of health information contained in these media to direct patients to trusted websites.
